# Effect of stress on insulin resistance among shift workers based on findings from the Korea National Health and Nutrition Examination Survey (2019, 2021): a retrospective cross-sectional study

**DOI:** 10.7586/jkbns.25.080

**Published:** 2026-02-23

**Authors:** Yea Seul Yoon, Wonhee Baek

**Affiliations:** 1 College of Nursing, CHA University, Pocheon, Korea; 2 College of Nursing, Gyeongsang National University, Jinju, Korea

**Keywords:** Insulin resistance, Metabolic syndrome, Occupational health, Shift work schedule, Stress, psychological

## Abstract

**Purpose:**

Insulin resistance is a major driver of metabolic disorders, and understanding factors associated with insulin resistance, particularly among Korean shift workers, is essential for the development of effective preventive strategies. This study aimed to identify factors related to insulin resistance using a complex-sample analysis of national survey data.

**Methods:**

In this retrospective cross-sectional study, secondary data were obtained from the 8th Korea National Health and Nutrition Examination Survey conducted in 2019 and 2021. All analyses were performed using a complex sampling design incorporating stratification, clustering, and sampling weights. Insulin resistance was assessed using the homeostatic model assessment for insulin resistance. Complex-sample multivariable logistic regression analyses were conducted to identify factors associated with insulin resistance.

**Results:**

The mean age of participants was 44.56 years, and 78.1% were men. Among the participants, 28.5% had insulin resistance. In the multivariable logistic regression analysis, higher body mass index was associated with increased odds of insulin resistance (odds ratio [OR] = 1.31, 95% confidence interval [CI] = 1.16~1.48). The presence of metabolic syndrome was associated with higher odds of insulin resistance compared with its absence (OR = 2.64, 95% CI = 1.31~5.33). Higher perceived stress was also associated with increased odds of insulin resistance (OR = 1.49, 95% CI = 1.04~2.14).

**Conclusion:**

Higher body mass index, metabolic syndrome, and elevated stress were associated with insulin resistance in Korean shift workers, highlighting the importance of managing both metabolic and psychological factors to reduce the risk of insulin resistance.

## INTRODUCTION

Insulin resistance is a pathological condition characterized by inadequate tissue response to normal insulin action, causing impaired glucose uptake and metabolism [1]. Insulin resistance affects approximately 26.5% of the global adult population [2], representing the central pathophysiological mechanism underlying Type 2 diabetes, and substantially increases the risk of cardiovascular diseases, obesity, hepatic steatosis, and metabolic syndrome [3]. This asymptomatic metabolic dysfunction precedes overt Type 2 diabetes by 10~15 years [1], during which progressive beta cell compensation masks deteriorating glucose homeostasis [4]. In Korea, the adult prevalence of insulin resistance is approximately 23.3% [2], while metabolic syndrome affected one-third of the adult population as of 2020, reflecting a 22.5% increase over the past two decades [5]. Insulin resistance remains clinically silent until complications manifest [6]; thus, identifying at-risk populations and modifiable predictors is critical for preemptive intervention.

Among the occupational factors that may influence insulin resistance risk, shift work has emerged as a significant exposure, affecting approximately 25% of the global workforce and many Korean workers across the manufacturing, healthcare, transportation, and service sectors [7,8]. Shift work disrupts the alignment between endogenous circadian rhythms and external environmental cues, causing chronic circadian discordance [9]. This desynchronization triggers multiple pathophysiological pathways that affect diverse health outcomes. At the metabolic level, disrupted suprachiasmatic nucleus signaling impairs vasopressin-mediated glucose uptake, while suppressed nocturnal melatonin secretion reduces pancreatic insulin synthesis [10]. Beyond glucose metabolism, circadian disruption dysregulates lipid metabolism, promotes systemic inflammation through elevated pro-inflammatory cytokines (TNF-α, IL-6), and impairs vascular endothelial function [11,12]. These mechanisms collectively increase susceptibility to insulin resistance, cardiovascular diseases, inflammatory disorders, and metabolic syndrome [13,14]. A recent meta-analysis of over 235,800 participants demonstrated that shift workers exhibited a 30% increased risk of Type 2 diabetes compared with the risk among day workers [13]. Additionally, an umbrella review has confirmed elevated risks of obesity (25% increase), hypertension (30% increase), and mental health disorders (32%~42% increase), establishing shift workers as a particularly vulnerable occupational group [14].

Previous research on insulin resistance in shift workers has revealed multiple potential risk factors. Demographic factors such as age and sex have shown varying associations, with some cohorts reporting an elevated insulin resistance risk among women shift workers, whereas others have suggested context-dependent patterns [2,15]. In addition, lifestyle factors, including poor dietary habits, physical inactivity, and irregular meal timing, are associated with a high prevalence of insulin resistance [16]. Metabolic parameters, particularly obesity and metabolic syndrome, demonstrate robust associations with insulin resistance; however, the temporal relationships remain debated [17]. Emerging evidence suggests that psychological stress contributes to the development of insulin resistance [18] and depressive symptoms have also been positively associated with increased insulin resistance [19]. Furthermore, quality of life, as a multidimensional indicator of physical and psychological well-being [20], was adjusted for to minimize confounding from general health status.

Although shift work is an independent risk factor for insulin resistance [16], previous studies on the relationship between shift work and metabolic disorders had several limitations [21,22]. Previous studies conducted primarily in the nursing and transportation sectors have largely focused on single occupations and predominantly single-sex samples [21,22]. These underscore the need for analyses that leverage nationally representative sampling frameworks and consider complex survey methods to generate robust population-level estimates [21,22]. This study extends this research trajectory by considering nationally representative data from Korean shift workers across diverse occupations.

This study aimed to identify significant predictors associated with the likelihood of having insulin resistance among Korean shift workers from the Korea National Health and Nutrition Examination Survey (KNHANES) [23]. To achieve this, we first characterized the study population by comparing clinical and lifestyle factors between shift workers with and without insulin resistance, followed by multivariable logistic regression analysis to determine independent associations.

## METHODS

### 1. Study design and population

In this retrospective cross-sectional study, we analyzed secondary data from the 8th KNHANES in 2019 and 2021. The KNHANES is a nationally representative cross-sectional survey designed to assess the health status, health behaviors, and dietary and nutritional intake of the Korean population. It provides fundamental data for establishing and evaluating the objectives of the National Health Promotion Comprehensive Plan and developing national health promotion programs. KNHANES collects data through health questionnaires, physical examinations, and nutritional surveys. The participants were selected using a two-stage stratified cluster sampling method based on population and housing census data.

In this study, participant records were retrieved from the 2019 and 2021 datasets of the 8th KNHANES, which included identical variables. From these datasets, we selected shift workers. In this study, shift workers were defined as individuals engaged in one of the following schedules: regular day-and-night shifts, 24-hour shifts, split shifts (working hours divided into two or more distinct periods in a single day), or irregular shifts. Participants diagnosed with diabetes and those with missing data on key variables were excluded. Among a total of 15,200 participants (8,110 in 2019 and 7,090 in 2021), we excluded individuals who were not shift workers (n = 14,808), those previously diagnosed with diabetes (n=35), and those with missing values for insulin resistance (n = 17). Consequently, 340 participants were included in this study.

### 2. Measures

#### 1) Participant characteristics

Sociodemographic and clinical variables included age, sex, education (high school or below and university or above), and household income quartile (low, lower-middle, upper-middle, and high).

#### 2) Lifestyle variables

Lifestyle characteristics included physical activity, smoking status, and alcohol consumption. Physical activity was assessed based on whether the participants met the weekly physical activity recommendations. Participants were classified as physically active if they engaged in at least 150 min of moderate-intensity activity per week, 75 min of vigorous-intensity activity per week, or an equivalent combination of both (with 1 min of vigorous activity considered equivalent to 2 min of moderate activity). The smoking status was categorized into nonsmokers (including former smokers) or current smokers. Alcohol consumption was classified as non-drinkers (those who had never consumed alcohol or drank less than once per month during the past year) or current drinkers (those who consumed alcohol at least once per month during the past year).

#### 3) Health-related variables

Depressive symptoms were defined as a binary variable (yes or no) based on whether participants had experienced feelings of sadness or despair for at least two consecutive weeks that interfered with daily activities during the previous year. Body mass index (BMI) was calculated by dividing body weight in kilograms by the square of height in meters (kg/m^2^). Participants were categorized into four groups based on BMI according to the Korean Society for the Study of Obesity Criteria: underweight (< 18.5 kg/m^2^), normal weight (18.5~22.9 kg/m^2^), overweight (23.0~24.9 kg/m^2^), and obese (≥ 25.0 kg/m^2^). Subjective health perception was assessed using a 5-point Likert scale ranging from 1 (very poor) to 5 (very good). Chronic diseases indicated the presence or absence of hypertension, diabetes, myocardial infarction, angina, osteoarthritis, osteoporosis, asthma, thyroid disease, and cancer. The presence or absence of depressive symptoms was classified based on whether the patient had a history of depression for > 2 consecutive weeks. Quality of life was assessed using the Health-Related Quality of Life Instrument with 8 Items [24]. This instrument comprises eight domains, including stair climbing, pain, and vitality, and each item is measured on a 4-point Likert scale ranging from “not at all” to “always.” The quality-of-life index was calculated using a formula derived from previous studies, with high scores indicating a high quality of life. Stress was measured on a 4-point Likert scale ranging from ‘I hardly feel it’ to ‘I feel it a lot,’ with high scores indicating pronounced stress levels.

#### 4) Metabolic syndrome

Metabolic syndrome was defined as the presence of three or more of the following according to the National Cholesterol Education Program Adult Treatment Panel Ⅲ: abdominal obesity (waist circumference ≥ 90 cm in men and ≥ 85 cm in women), elevated blood pressure (systolic ≥ 130 mmHg or diastolic ≥ 85 mmHg), low levels of high-density lipoprotein cholesterol (< 40 mg/dL in men and < 50 mg/dL in women), high triglyceride levels (≥ 150 mg/dL), and high fasting glucose levels (≥ 100 mg/dL) [25].

#### 5) Insulin resistance

Insulin resistance was calculated using the homeostatic model assessment for insulin resistance (HOMA-IR) formula. HOMA-IR has been validated in multiple studies as a reliable surrogate marker of insulin resistance [26].

The HOMA-IR formula is as follows:

HOMA-IR = [fasting insulin (µU/mL) × fasting glucose (mg/L)] / 405

In this study, differential HOMA-IR cut-off values were applied based on sex and physiological status (menopausal status) to classify participants as having normal insulin sensitivity or insulin resistance: 2.20 for men, 2.55 for premenopausal women, and 2.03 for postmenopausal women [26]. Participants with HOMA-IR values below these thresholds were classified as having normal insulin sensitivity, while those with values at or above the thresholds were classified as having insulin resistance.

### 3. Statistical methods

All statistical analyses were performed considering the complex sampling design of the KNHANES, including stratification, clustering, and sampling weights. To ensure national representativeness for the combined survey periods, integrated sampling weights were calculated by averaging the annual weights from 2019 and 2021. To handle strata with a single sampling unit, the 'lonely primary sampling unit' adjustment was applied to ensure the stability of variance estimation. To minimize bias in variance estimation and maintain the integrity of the complex sampling design, missing values were not excluded listwise but were treated as valid non-responses (valid subpopulation) within the complex sample analysis.

Descriptive statistics for the participants were analyzed using complex sample estimates. Categorical variables were presented as unweighted frequencies and weighted percentages, while continuous variables were presented as weighted means and standard errors. Differences in continuous variables between participants with and without insulin resistance were analyzed using complex-sample t-tests, and differences in categorical variables were examined using complex-sample Pearson chi-square tests with Rao and Scott adjustments. To identify significant predictors associated with the likelihood of having insulin resistance, complex-sample multivariable logistic regression analyses were performed.

All statistical analyses were conducted using R software (Version 4.3.1; R Foundation for Statistical Computing, Vienna, Austria). For bivariate analyses, statistical significance was assessed using two-sided *p*-values, with *p* < .050 considered statistically significant. For multivariable logistic regression analyses, results were presented as odds ratios with 95% confidence intervals (CIs), and statistical significance was determined when the 95% CI did not include 1.

### 4. Ethical considerations

This study was approved by the Institutional Review Board of Gyeongsang National University (Approval number: GIRB-D25-NX-0123), and written informed consent was waived.

## RESULTS

### 1. Participant characteristics

The demographic and clinical characteristics of the participants are presented in Table 1. The complex-sample analysis indicated that the mean age of the participants was 44.56 (standard error [SE] = 1.00) years. Most participants were men (78.1%), and 54.6% had a final education level of high school or below. Most participants were in the highest (fourth) household income quartile (42.9%). Regarding lifestyle factors, 51.4% of participants did not engage in physical activity, 73.4% were nonsmokers, and 72.8% consumed alcohol. Concerning health status, the mean BMI was 24.27 (SE = 0.22) and 36.4% of participants were classified as obese. The average score for subjective health perception was 3.34 (SE = 0.05), and 95.3% reported no depressive symptoms within the past 2 weeks. Furthermore, 21.1% of the participants had metabolic syndrome, and 15.1% had hypertension. The prevalence of myocardial infarction or angina was 1.2%, while those of degenerative arthritis, osteoporosis, asthma, thyroid disease, and cancer were 4.4%, 1.1%, 1.1%, 1.3%, and 1.4%, respectively. The mean quality of life score was 0.84 (SE = 0.01), and the mean stress score was 2.01 (SE = 0.04). The mean HOMA-IR value was 2.06 (SE = 0.11). Regarding insulin resistance, 71.5% of participants were classified as having normal insulin sensitivity, whereas 28.5% had insulin resistance. The occupational characteristics of the participants are presented in Appendix 1. Based on the Korean Standard Classification of Occupations, plant and machine operators and assemblers accounted for the largest proportion at 25.8%. Regarding shift work patterns, 59.9% of the participants were engaged in regular day-and-night shift work.

### 2. Differences in insulin resistance according to participant characteristics

As shown in Table 2, sex, BMI, subjective health perception, metabolic syndrome, and stress were significantly associated with insulin resistance. The prevalence of insulin resistance was higher in men than in women (*p* = .034). Participants with insulin resistance had a significantly higher BMI than did those in the normal insulin sensitivity group (*p* < .001). Participants with insulin resistance reported significantly lower subjective health perception compared to the normal insulin sensitivity group (*p* = .001). Participants with metabolic syndrome showed a more pronounced insulin resistance than did those without metabolic syndrome (*p* < .001). In addition, participants with high stress levels had marked insulin resistance (*p* = .004).

### 3. Factors associated with insulin resistance among shift workers: multivariate logistic regression analysis

Results of the multivariable logistic regression analysis are presented in Table 3. After adjusting for potential confounders, BMI, metabolic syndrome, and psychological stress were independently associated with insulin resistance. Specifically, BMI was analyzed as a continuous variable, and each 1 kg/m^2^ increase in BMI was associated with a 31% increase in the odds of insulin resistance (β = 0.27, odds ratio [OR] = 1.31, 95% CI = 1.16~1.48). Participants with metabolic syndrome had significantly higher odds of insulin resistance compared with those without metabolic syndrome (β = 0.97, OR = 2.64, 95% CI =1.31~5.33). Psychological stress, measured on a 4-point Likert scale and treated as a continuous variable, was also independently associated with insulin resistance; each 1-point increase in stress score was associated with a 49% increase in the odds of insulin resistance (β = 0.40, OR = 1.49, 95% CI =1.04~2.14).

## DISCUSSION

We investigated the prevalence of insulin resistance and its predictors among Korean shift workers using nationally representative KNHANES data and a complex sampling methodology [23]. Weighted logistic regression models were used to identify independent predictors while adjusting for relevant covariates. The prevalence of insulin resistance among shift workers in this study was 28.5%, higher than the global and Korean adult averages of 26.5% and 23.3%, respectively [2]. While these findings should be interpreted cautiously, considering the variability in insulin resistance cut-offs, sex distribution, and menopausal status across studies [2], and the absence of a non-shift worker comparison group in our study, they may reflect potential metabolic vulnerabilities in this occupational population. Three independent predictors emerged: higher BMI, metabolic syndrome, and elevated perceived stress were associated with increased insulin resistance risk.

BMI remained a significant predictor after adjusting for metabolic syndrome, indicating that total adiposity contributes to insulin resistance through pathways not fully captured by metabolic syndrome criteria. While metabolic syndrome defines abdominal obesity by waist circumference thresholds, BMI reflects total fat mass including subcutaneous depots that secrete adipokines and inflammatory mediators [1,16]. In shift workers, this relationship may be influenced by circadian and behavioral factors associated with shift work, including disrupted sleep-wake cycles and altered meal timing patterns [10,27]. However, our study did not assess specific shift work characteristics (such as shift type, rotation patterns, or night shift frequency), precluding definitive conclusions about how particular shift work schedules might modify the BMI-insulin resistance relationship. BMI's practical advantage lies in its simplicity, enabling self-monitoring without laboratory resources despite healthcare access barriers faced by shift workers.

Metabolic syndrome demonstrated the strongest association (OR = 2.64), remaining significant after BMI adjustment. This pronounced effect likely reflects synergistic interactions among syndrome components rather than simple additive effects. Each component—hypertension, dyslipidemia, hyperglycemia, and abdominal obesity—not only indicates existing dysfunction but also actively promotes insulin resistance through distinct pathways: endothelial dysfunction impairing insulin-mediated glucose delivery, ectopic lipid deposition in muscle and liver, and glucotoxicity further impairing insulin signaling [16,24]. Meta-analyses demonstrate that shift work increases metabolic syndrome prevalence [24], though the specific mechanisms linking shift work schedules to metabolic syndrome development require further investigation with detailed exposure characterization. However, metabolic syndrome assessment requires comprehensive clinical and laboratory evaluations, limiting feasibility for frequent monitoring.

The independent contributions of BMI and metabolic syndrome suggest they capture different dimensions of metabolic risk. BMI primarily reflects quantitative adipose burden and inflammatory state, while metabolic syndrome indicates qualitative dysregulation across multiple organ systems. For practical surveillance, BMI monitoring can facilitate frequent self-assessment, while periodic comprehensive metabolic syndrome evaluation should be conducted regardless of BMI status, as metabolic abnormalities may occur even with normal weight. Individuals with either elevated BMI or any metabolic syndrome components warrant intensified monitoring. Longitudinal study demonstrates bidirectional relationships, in which insulin resistance can promote subsequent weight gain and metabolic dysfunction [28]. However, our study lacks detailed shift work exposure data (shift patterns, duration, night shift frequency) and a non-shift worker comparison group, precluding definitive conclusions about shift work's specific contribution to the observed associations.

Elevated perceived stress was independently associated with insulin resistance, with a 49% increase in the odds among shift workers reporting higher stress levels. This is consistent with previous evidence demonstrating an association between perceived stress and metabolic dysfunction [19,21]. A meta-analysis of 17 studies showed that self-reported perceived stress was significantly correlated with visceral obesity and lipid parameters of metabolic syndrome [29]. Among shift workers, elevated perceived stress is associated with metabolic syndrome and altered cortisol patterns [30,31]. Notably, perceived stress represents an individual’s subjective appraisal of stress rather than objective stressors or biological markers [32]. While chronic stress induces insulin resistance through hypothalamic-pituitary-adrenal axis activation [13,19] and inflammatory pathways [16,19], perceived stress reflects complex interactions between external demands, coping resources, and psychological appraisal [32].

Sex was not a significant predictor of insulin resistance in the multivariate model (OR = 0.61, *p* = .208), contrasting with univariate findings where men showed higher insulin resistance prevalence than women (*p* = .034). This change from bivariate significance to multivariable non-significance occurred after adjusting for BMI, suggesting that sex differences in insulin resistance may be largely mediated by body composition rather than representing an independent sex effect. When BMI was included in the model, the apparent protective effect of women was attenuated, indicating that lower insulin resistance prevalence among women in our sample may primarily reflect differences in adiposity rather than sex-specific metabolic mechanisms.

This finding appears to contradict previous evidence suggesting metabolic vulnerabilities in women shift workers [13,14]. However, most meta-analyses have focused on diabetes incidence rather than insulin resistance [13,21,22], and the progression from insulin resistance to overt diabetes may be influenced by sex-specific factors including beta cell function, hormonal fluctuations, and compensatory mechanisms [33,34]. Several contextual factors warrant consideration. First, occupational sampling characteristics differ substantially across studies: previous research has predominantly examined women-dominated nursing cohorts [22,35] characterized by moderate-to-low-intensity physical activity combined with high cognitive demands, which may create distinct metabolic risk profiles compared with men-dominated industries such as transportation and manufacturing [36]. Our sample (78.1% men) likely reflects different occupational exposures. Second, cross-sectional analyses may underrepresent metabolically vulnerable workers who opt out of shift work schedules due to health challenges [21,22]. Third, the predominantly men composition may have limited statistical power to detect sex-specific interactions. These findings suggest that the relationship between sex and insulin resistance in shift workers is highly context-dependent, potentially reflecting unique occupational, body composition, and methodological factors in our Korean sample.

Physical activity did not emerge as a significant predictor of insulin resistance in our analysis, despite well-established evidence that regular physical activity improves insulin sensitivity through multiple mechanisms including enhanced glucose uptake, improved mitochondrial function, and reduced systemic inflammation [37,38]. This null finding likely reflects our study population characteristics and cross-sectional design features. Over half of our sample (51.7%) worked in manual labor occupations, whose high baseline occupational physical activity may have created a ceiling effect, diminishing the measurable additional metabolic benefit of leisure-time exercise. Additionally, our binary assessment of leisure-time physical activity did not capture total physical activity exposure (occupational plus leisure-time), which may be particularly important in populations with high occupational activity. This measure also did not differentiate between occupational and leisure-time physical activity—which have distinct metabolic effects [39]—nor did it capture variations in exercise intensity, duration, type, or consistency that influence insulin sensitivity. The cross-sectional design cannot assess temporal patterns such as exercise consistency or duration of physical activity habits, both critical for metabolic adaptations. We initially considered converting physical activity data to metabolic equivalents (METs) for more precise quantification, but substantial missing data and logical inconsistencies in the activity frequency and duration variables would have compromised the validity of such calculations. Therefore, we maintained the binary physical activity variable despite its limitations. Future research employing objective physical activity measures (such as accelerometry) and detailed assessments distinguishing occupational from leisure-time activity may better elucidate the relationship between physical activity patterns and insulin resistance in shift workers.

Cancer and arthritis showed no significant association with insulin resistance, likely reflecting the subclinical nature of insulin resistance in our relatively young working population and insufficient exposure duration for chronic disease development [4,6,40]. Insulin resistance progresses through an extended asymptomatic phase, during which affected individuals may report good health despite objective metabolic dysfunction [4]. Studies have demonstrated that insulin resistance is associated with subclinical vascular changes and precedes symptomatic disease by years, dissociating laboratory-detected abnormalities from perceived health status [40]. These findings underscore that the absence of health complaints does not exclude insulin resistance, supporting the rationale for objective metabolic screening in high-risk occupational populations.

This study has several notable strengths, including the use of the nationally representative KNHANES data with a complex sampling methodology, which ensures generalizability to Korean shift workers across diverse occupations [23]. We included multiple occupational sectors and used weighted multivariable logistic regression analyses to account for the complexity of the survey design. Most notably, the inclusion of metabolic syndrome as a comprehensive covariate allowed us to effectively control for the intricate and overlapping influences of individual metabolic components on insulin resistance. The use of HOMA-IR has enabled practical assessment in a large epidemiological study [1].

Nevertheless, this study has some significant limitations. The cross-sectional design precludes causal inferences and establishment of temporal relationship establishment between predictors and insulin resistance. The absence of a non-shift worker comparison group limits our ability to determine whether the observed 28.5% prevalence—higher than Korean adult averages—specifically results from shift work. Future comparative studies with detailed shift work exposure characterization (shift type, duration, rotation patterns, cumulative exposure) are needed to clarify independent metabolic effects. Our assessment of perceived stress relied on a single-item self-report measure that may not capture multidimensional stress constructs and is subject to recall bias. Quality-of-life instruments may have insufficient sensitivity for detecting subclinical metabolic dysfunction. Additionally, self-reported cancer and arthritis data may have underestimated the true prevalence. Notably, KNHANES lacked information on chronic liver and kidney diseases, both of which can significantly influence metabolic status and insulin resistance [41]. Future studies should incorporate comprehensive liver and kidney function screening. Physical activity assessment was limited to binary self-report measures that inadequately capture activity complexity, intensity variations, or occupational versus leisure-time distinctions. Data quality issues prevented sophisticated MET-based quantification. Residual confounders from unmeasured variables—detailed dietary patterns, sleep quality/duration, specific shift schedules, and shift work duration—may exist. Important metabolic risk factors including nutritional intake and sleep duration were not assessed due to data availability constraints in KNHANES: sleep assessment instruments differed between 2019 and 2021, and nutritional data lacked consistency across survey years, preventing meaningful cross-year integration. This reflects inherent characteristics of secondary data analysis using previously collected survey data, where variable selection is bound by available measures rather than prospective study design. Finally, findings may have limited generalizability beyond Korean populations, and the predominantly men sample may limit sex-specific insights.

## CONCLUSION

This nationally representative study identified insulin resistance prevalence of 28.5% among Korean shift workers, with higher BMI, metabolic syndrome, and elevated perceived stress as independent predictors. The independent effects of BMI and metabolic syndrome underscore that both general adiposity and metabolic dysregulation warrant clinical attention, suggesting practical surveillance strategies: routine BMI monitoring for accessible self-assessment and periodic comprehensive metabolic syndrome evaluation regardless of BMI status. The significant association between perceived stress and insulin resistance highlights the importance of addressing psychological factors alongside metabolic risk. However, important limitations must be acknowledged: the absence of detailed shift work exposure data and a non-shift worker comparison group preclude definitive conclusions about shift work's specific contribution, while the cross-sectional design limits causal inferences. Future longitudinal studies with detailed shift work characterization, comparison groups, objective physical activity measurement, and comprehensive health screening are needed to clarify causal pathways and develop targeted interventions for this occupational population.

## Figures and Tables

**Table 1.** Demographic and Clinical Characteristics of Participants (N = 340)

Variables	Categories	N (%)	M ± SE
Age (years)			44.56 ±1.00
Sex	Men	255 (78.1)	
	Women	85 (21.9)	
Final education	High school or below	200 (54.6)	
	University or above	140 (45.4)	
Household income quartile	Low	13 (3.4)	
	Lower-middle	79 (22.0)	
	Upper-middle	104 (31.8)	
	High	143 (42.9)	
Physical activity	No	169 (51.4)	
	Yes	170 (48.6)	
Smoking status	No	253 (73.4)	
	Yes	87 (26.6)	
Alcohol consumption	No	99 (27.2)	
	Yes	241 (72.8)	
Depressive symptoms	No	323 (95.3)	
	Yes	17 (4.7)	
Body mass index (kg/m^2^)			24.27 ± 0.22
	Underweight (< 18.5)	8 (3.3)	
	Normal weight (18.5~22.9)	119 (35.0)	
	Overweight (23.0~24.9)	87 (25.4)	
	Obese (≥ 25.0)	126 (36.4)	
Subjective health perception			3.34 ± 0.05
Metabolic syndrome	No	266 (78.9)	
	Yes	72 (21.1)	
Hypertension	No	269 (84.9)	
	Yes	71 (15.1)	
Myocardial infarction or angina	No	334 (98.8)	
	Yes	6 (1.2)	
Degenerative arthritis	No	322 (95.6)	
	Yes	18 (4.4)	
Osteoporosis	No	334 (98.9)	
	Yes	6 (1.1)	
Asthma	No	337 (98.9)	
	Yes	3 (1.1)	
Thyroid disease	No	334 (98.7)	
	Yes	6 (1.3)	
Cancer	No	333 (98.6)	
	Yes	7 (1.4)	
Quality of life			0.84 ± 0.01
Stress			2.01 ± 0.04
HOMA-IR			2.06 ± 0.11
Insulin resistance	No	243 (71.5)	
	Yes	97 (28.5)	

Values are presented as unweighted n (weighted %) or weighted mean ± standard error. Missing values were treated as valid non-responses in the complex sample analysis. Household income quartile, physical activity, and metabolic syndrome had missing values (n = 1, 1, and 2, respectively). M= Mean; SE= Standard error; HOMA-IR = Homeostatic model assessment for insulin resistance.

**Table 2.** Differences in Weighted Population Estimates According to Insulin Resistance Status (N = 340)

Variables	Categories	Normal insulin sensitivity (n = 243, 71.5%)	Insulin resistance (n = 97, 28.5%)	t or F	*p*
		n (%) or M ± SE			
Age		45.06 ± 1.15	43.34 ± 1.70	0.86	.390
Sex	Men	176 (75.1)	79 (85.5)	4.57	.034
	Women	67 (24.9)	18 (14.5)		
Final education	High school or below	144 (55.3)	56 (52.7)	0.14	.707
	University or above	99 (44.7)	41 (47.3)		
Household income quartile	Low	7 (2.6)	6 (5.2)	0.58	.629
	Lower-middle	57 (23.2)	22 (18.9)		
	Upper-middle	74 (30.8)	30 (34.0)		
	High	104 (43.3)	39 (41.9)		
Body mass index (kg/m^2^)		23.26 ± 0.22	26.80 ± 0.41	-7.46	< .001
Physical activity	No	118 (49.2)	51 (56.9)	1.42	.235
	Yes	125 (50.8)	45 (43.1)		
Smoking status	No	182 (73.9)	71 (72.2)	0.08	.780
	Yes	61 (26.1)	26 (27.8)		
Alcohol consumption	No	69 (26.0)	30 (30.2)	0.44	.510
	Yes	174 (74.0)	67 (69.8)		
Depression	No	231 (96.0)	92 (93.8)	0.62	.433
	Yes	12 (4.0)	5 (6.2)		
Subjective health perception		3.43 (0.05)	3.12 (0.08)	-3.27	.001
Metabolic syndrome	No	213 (88.4)	53 (54.9)	39.04	< .001
	Yes	29 (11.6)	43 (45.1)		
Hypertension	No	198 (86.9)	71 (80.1)	2.21	.139
	Yes	45 (13.1)	26 (19.9)		
Myocardial infarction or angina	No	239 (99.0)	95 (98.4)	0.71	.401
	Yes	4 (1.0)	2 (1.6)		
Degenerative arthritis	No	228 (95.1)	94 (96.8)	0.53	.469
	Yes	15 (4.9)	3 (3.2)		
Osteoporosis	No	240 (99.3)	94 (98.1)	1.46	.229
	Yes	3 (0.7)	3 (1.9)		
Asthma	No	242 (99.1)	95 (98.2)	0.35	.554
	Yes	1 (0.9)	2 (1.8)		
Thyroid disease	No	238 (98.7)	96 (98.9)	0.02	.891
	Yes	5 (1.3)	1 (1.1)		
Cancer	No	236 (98.0)	97 (100.0)	2.1	.150
	Yes	7 (2.0)	0 (0.0)		
Quality of life		0.85 ± 0.01	0.83 ± 0.01	1.15	.253
Stress		1.99 ± 0.05	2.23 ± 0.07	-2.97	.004

Values are presented as unweighted n (weighted %) or weighted mean ± standard error. Household income quartile, physical activity, and metabolic syndrome had missing values (n = 1, 1, and 2, respectively). All *p*-values were calculated using the complex-sample t-test or Rao and Scott adjusted F statistics. M=Mean; SE=Standard error.

**Table 3.** Factors Associated with Insulin Resistance among Shift Workers: Multivariate Logistic Regression Analysis

Factors	β	OR	*p*	95% CI
Sex				
Women	-0.49	0.61	.208	0.29~1.31
Men	Ref			
Body mass index	0.27	1.31	< .001	1.16~1.48
Subjective health perception	-0.39	0.67	.077	0.44~1.04
Metabolic syndrome				
Yes	0.97	2.64	.007	1.31~5.33
No	Ref			
Stress	0.40	1.49	.032	1.04~2.14

Body mass index, perceived stress, and subjective health perception were treated as continuous variables. Odds ratios indicate changes in odds per one-unit increase. Body mass index was scaled per 1 kg/m^2^; perceived stress and subjective health perception were measured using 4-point and 5-point scales, respectively. OR = Odds ratio; CI = Confidence interval; Ref = Reference.

## Data Availability

The data used in this study are publicly available and can be accessed through the Korea Open Data Portal (https://www.data.go.kr).

## Appendix 1. Occupational Characteristics

**Table:**

Variables	Categories	Non-weighted sample size (weighted %)
^†^Type of work	Managers	1 (0.3)
	Professional and related workers	39 (14.8)
	Clerical workers	13 (3.5)
	Service workers	64 (19.2)
	Sales workers	9 (2.9)
	Skilled agricultural, forestry and fishery workers	9 (2.1)
	Craft and related trades workers	19 (7.2)
	Plant and machine operators and assemblers	86 (25.8)
	Elementary occupations	71 (16.6)
	Armed forces personnel	1 (0.2)
Type of work schedule	Regular day-and night shift work	192 (59.9)
	24-hour shift work	73 (18.0)
	Split shift work	39 (10.4)
	Irregular shift work	36 (11.7)

† There were missing values (n = 28).
